# Wise Practices for Cultural Safety in Electronic Health Research and Clinical Trials With Indigenous People: Secondary Analysis of a Randomized Clinical Trial

**DOI:** 10.2196/14203

**Published:** 2019-11-04

**Authors:** Marion A Maar, Valerie Beaudin, Karen Yeates, Lisa Boesch, Peter Liu, Kian Madjedi, Nancy Perkins, Diane Hua-Stewart, Faith Beaudin, Mary Jo Wabano, Sheldon W Tobe

**Affiliations:** 1 Faculty of Medicine Northern Ontario School of Medicine Laurentian University Sudbury, ON Canada; 2 M'Chigeeng Health Centre M'Chigeeng First Nation, ON Canada; 3 Department of Medicine Queen's University Kingston, ON Canada; 4 Department of Research Northern Ontario School of Medicine Sudbury, ON Canada; 5 University of Ottawa Heart Institute Ottawa, ON Canada; 6 Department of Medicine Northern Ontario School of Medicine Sudbury, ON Canada; 7 Department of Medicine Sunnybrook Health Sciences Centre, Sunnybrook Research Institute University of Toronto Toronto, ON Canada; 8 Naandwechige-Gamik Health Centre Wiikwemkoong Unceded Territory, ON Canada

**Keywords:** mobile health, process evaluation, implementation science, Indigenous peoples, health care texting, SMS, hypertension, task shifting, community-based participatory research, DREAM-GLOBAL

## Abstract

**Background:**

There is a paucity of controlled clinical trial data based on research with Indigenous peoples. A lack of data specific to Indigenous peoples means that new therapeutic methods, such as those involving electronic health (eHealth), will be extrapolated to these groups based on research with other populations. Rigorous, ethical research can be undertaken in collaboration with Indigenous communities but requires careful attention to culturally safe research practices. Literature on how to involve Indigenous peoples in the development and evaluation of eHealth or mobile health apps that responds to the needs of Indigenous patients, providers, and communities is still scarce; however, the need for community-based participatory research to develop culturally safe technologies is emerging as an essential focus in Indigenous eHealth research. To be effective, researchers must first gain an in-depth understanding of Indigenous determinants of health, including the harmful consequences of colonialism. Second, researchers need to learn how colonialism affects the research process. The challenge then for eHealth researchers is to braid Indigenous ethical values with the requirements of good research methodologies into a culturally safe research protocol.

**Objective:**

A recent systematic review showed that Indigenous peoples are underrepresented in randomized controlled trials (RCTs), primarily due to a lack of attention to providing space for Indigenous perspectives within the study frameworks of RCTs. Given the lack of guidelines for conducting RCTs with Indigenous communities, we conducted an analysis of our large evaluation data set collected in the Diagnosing Hypertension-Engaging Action and Management in Getting Lower Blood Pressure in Indigenous Peoples and Low- and Middle- Income Countries (DREAM-GLOBAL) trial over a period of five years. Our goal is to identify wise practices for culturally safe, collaborative eHealth and RCT research with Indigenous communities.

**Methods:**

We thematically analyzed survey responses and qualitative interview/focus group data that we collected over five years in six culturally diverse Indigenous communities in Canada during the evaluation of the clinical trial DREAM-GLOBAL. We established themes that reflect culturally safe approaches to research and then developed wise practices for culturally safe research in pragmatic eHealth research.

**Results:**

Based on our analysis, successful eHealth research in collaboration with Indigenous communities requires a focus on cultural safety that includes: (1) building a respectful relationship; (2) maintaining a respectful relationship; (3) good communication and support for the local team during the RCT; (4) commitment to co-designing the innovation; (5) supporting task shifting with the local team; and (6) reflecting on our mistakes and lessons learned or areas for improvement that support learning and cultural safety.

**Conclusions:**

Based on evaluation data collected in the DREAM-GLOBAL RCT, we found that there are important cultural safety considerations in Indigenous eHealth research. Building on the perspectives of Indigenous staff and patients, we gleaned wise practices for RCTs in Indigenous communities.

**Trial Registration:**

ClinicalTrials.gov NCT02111226; https://clinicaltrials.gov/ct2/show/NCT02111226

## Introduction

### Background

There is a paucity of controlled clinical trial data based on research with Indigenous peoples. As health care moves toward the era of personalized medicine, a lack of data specific to Indigenous peoples will mean that new therapeutic methods will have to be extrapolated to Indigenous patients based on work with other populations, or that new therapies, such as those involving eHealth, will not be applicable. Appropriate, ethical, and culturally safe research can be undertaken with Indigenous communities but requires attention to good research practices that improve the overall quality of the research effort.

### Engaging With Indigenous Peoples in Electronic Health

The literature shows that a participatory development process involving patient and provider end users, as well as system stakeholders, is essential to optimize the uptake of electronic health (eHealth) technologies and to support sustainable innovations in health care [1,2]. Using participatory approaches, researchers should be able to explain how their results can positively impact the communities involved in the studies. Moreover, Van Gemert-Pijnen and colleagues advocate for a holistic approach to eHealth development that empathizes the importance of understanding the intervention as a whole and “takes into account the complexity of health care and the rituals and habits of patients and other stakeholders” [2].

In other words, researchers describe the need to understand the culture of the organizations that are touched and, most importantly, the culture of the people whose life is altered in some way by the technological innovation. There are many lay definitions of culture, but for the purpose of incorporating the concept of culture into eHealth research it is useful to adopt a definition from anthropology, the field where culture has been formally studied as the “mental and physical reactions and activities that characterize the individuals of a social group” [3].

Deconstructing the role of culture is key for the success of eHealth innovations, and it is particularly essential when an innovation is planned in nonmainstream cultural contexts such as Indigenous communities.

Engaging Indigenous communities in research is possible when the research question is of current interest to the communities. When researchers are aware of emerging health issues and eHealth or mobile health (mHealth) apps that have not yet caught the attention of the general population, it is much more challenging to conduct collaborative research. Literature on how to involve Indigenous peoples in the development and evaluation of eHealth/mHealth apps in order to respond to the needs of Indigenous patients, providers, and communities is still scarce; however, the need for community-based participatory research to develop culturally safe technologies is emerging as an essential focus in Indigenous eHealth research [4-6]. To our knowledge, concrete deconstructions of the factors that affect Indigenous eHealth innovations, such as: (1) the complexity of the Indigenous health system; (2) the unique historic, jurisdictional, and geographic issues; (3) effective methods of cocreation of interventions that reflect the diversity of Indigenous cultures; and (4) the Indigenous patient, provider, community, and organizational needs to facilitate uptake; have not been previously published [6]. To evaluate eHealth innovation from these highly relevant perspectives, researchers must adopt appropriate Indigenous research approaches [4,7,8].

### Evaluating Electronic Health Interventions

The Randomized Controlled Trial (RCT) is widely considered to be the gold standard research methodology for evaluating whether a cause-and-effect relationship between an intervention and an outcome may exist. This traditional model of an RCT is known as the explanatory RCT and assesses outcomes under optimally controlled conditions, minimizing bias and controlling for potential confounders. One major criticism of this model of RCT is its intrinsic limitation in accommodating real-life practice conditions, such as the diversity of participants, settings, or human factors; however, these complex variables exist and interact in eHealth interventions [9,10].

Pragmatic RCTs offer a methodology and research design which prioritizes the evaluation of effectiveness (the performance of the intervention in real-world conditions) over efficacy (whether an intervention works under ideal, controlled circumstances). In a pragmatic trial design, the same measures of effectiveness can be used as in an explanatory RCT, however, the intervention is tested in the context of everyday practice settings, which in turn requires a rigorous analysis of complex variables reflective of real-life conditions [11]. A pragmatic trial can provide data on the uptake and sustainability of technological innovations because it accommodates the study of key implementation factors in the everyday environments of clinics and communities. Process evaluations can be incorporated into a pragmatic trial, to study implementation conditions which can clarify why different outcomes exist for the same intervention at different sites.

In Indigenous communities, context includes diversity in geography, policy, and culture of patients, providers, and the organization, which all play an important role in implementation [7,12]. Analyzing these factors makes the results of pragmatic trials more informative to other Indigenous communities.

### Preparing for Electronic Health Research with Indigenous Communities

If an innovation is studied as part of a clinical research trial, then rigorous data collection and analysis put considerable demands on researchers and health care workers [13]. Adding to this workload, researchers require cultural and historical training to become ready to collaborate with Indigenous communities.

First, to be effective, researchers must gain an in-depth understanding of Indigenous determinants of health in the participating population. Indigenous health is heavily impacted by the ongoing harmful consequences of colonialism; in Canada this includes the multi-generational effects of the residential school abuses [14], rampant adverse childhood experiences [15-17], loss of life styles and language [18], and the dispossession of traditional lands, resulting in food insecurity [19], collapse of traditional economies, and increase in chronic disease [20-24]. Today, health disparities in Indigenous communities continue to be reinforced through social exclusion, discrimination, and systemic racism in health care and society overall [25].

Second, researchers need to learn how colonialism affects the research process. Willie Ermine, an Indigenous ethics scholar, states that:

One of the festering irritants for Indigenous peoples, in their encounter with the West, is the brick wall of a deeply embedded belief and practice of Western universality [26].

This belief manifests in a “researcher knows best” [27] attitude with research relationships that deteriorate from problematic to antagonistic:

Outside experts, often with little knowledge of the realities of Aboriginal community life, were commonly in a position where they controlled all aspects of the Aboriginal research projects. These experts decided which research questions warranted investigation, which methods should be used to collect data, and how the data should be interpreted and disseminated. The resulting research projects gave little consideration to the insider perspective of Aboriginal community members, existing Indigenous knowledge, the cultural competence of the research methods used, or to collaborative interpretations. Data and results were rarely accessible to community members. Knowledge transfer strategies geared to support community action on a particular problem were absent. Commonly, at the end of a project, outside experts would recommend inappropriate or unworkable solutions to community problems [28].

For decades, government and academic research have largely failed to improve Indigenous health [28]. Research relationships have been marred by disrespect for Indigenous world views, and Indigenous communities have experienced broken trust and unethical and oppressive conduct [26,29,30]. For many Indigenous leaders, research, when conducted by outsiders, has become a dirty word [31]. The consequence of the historic practice of using research as a vehicle for hegemony manifests today as distrust towards health research even when the research need is identified by Indigenous communities. To protect communities from harm, there is a growing movement within Indigenous circles to conduct research autonomously, based on community-perceived desires and needs [29,32].

However, researchers can establish relationships if they are committed to a community-based participatory approach [8,33], building a trust-based relationship, creating ethical space for dialogue [7,26], and respecting Indigenous culture and worldviews [32,34]. Researchers need sensitivity training supported by cultural immersion to learn protocols and traditions before they can support a culturally relevant research process [35]. The challenge then for eHealth researchers is to braid Indigenous ethical values with the requirements of good research methodologies into a culturally safe research protocol.

### Collaborating With Indigenous Peoples on Randomized Clinical Trials in Electronic Health

Many researchers are unable to create an ethical space for Indigenous perspectives within the study frameworks of RCTs, which in turn prevents them from building productive research relationships with Indigenous communities. A recent systematic review showed that Indigenous peoples are indeed underrepresented in RCTs [36]. The authors suggest that:

Rather than sidestepping Aboriginal communities, researchers should consider participatory methods for conducting RCTs with Aboriginal communities to increase the cultural relevance of these designs and to enhance the process of implementation of RCTs for optimal recruitment, engagement and retention of participants in trials, while being sensitive to the social value and cultural traditions of Aboriginal communities [36].

We found that an explanatory RCT approach to eHealth in Indigenous community contexts was incongruent with Indigenous epistemologies. Instead, we created a pragmatic RCT protocol for DREAM-GLOBAL. The RCT protocol was shaped by our formative implementation research with Indigenous communities using the Intervention and Research Readiness Engagement and Assessment of Community Health Care (I-RREACH) community engagement tool. We strove to allow enough flexibility to address the unique circumstances of each of the Indigenous communities from their diverse cultural perspectives [7]. This approach helped us to build the community-researcher relationship and supported discovery in our cross-cultural research.

### The DREAM-GLOBAL Clinical Trial in Indigenous Communities in Canada

DREAM-GLOBAL is a complex mHealth intervention and pragmatic RCT designed to achieve improvements in blood pressure (BP) control in low resource environments using evidence of hypertension guidelines. Briefly, the objective in Canada was to evaluate the effectiveness of an innovative mHealth program using SMS text messages and electronic transfer of BP measures from patients to providers on BP control of Indigenous peoples [12]. DREAM-GLOBAL requires changes in the way services are provided that affect patients, providers, and the local health system [37].

DREAM-GLOBAL demonstrated that innovations in health services delivery, mHealth technologies, and patient engagement could be successfully implemented in collaboration with Indigenous communities in Canada [38]. In an anonymous survey designed to monitor patients’ satisfaction with the intervention, 98% of the Indigenous participants (n=165/169) stated they would recommend the DREAM-GLOBAL program to a friend or relative [38]. We consider this statistic, combined with successful trial completion in all partner communities, as a preliminary indication of our culturally appropriate approach. However, given the lack of guidelines for conducting RCTs with Indigenous communities [36] it is important to critically evaluate our approach and to share our learning to support the future development of culturally safe, collaborative eHealth and RCT research within diverse Indigenous communities.

In this paper, we analyze the large qualitative data set from our pragmatic RCT collected in six culturally diverse Indigenous communities over a period of five years to identify culturally safe research practices in pragmatic RCTs.

## Methods

### Data Sources

The DREAM-GLOBAL RCT was implemented in six First Nations communities, representing Cree, Anishinabek (Odawa, Ojibwa, and Potawatomi) and Mi'kmaq tribes in remote northern, rural, and periurban locations in three Canadian provinces. Trial participants self-identified as Indigenous, and further mostly as First Nations with legal Indian Status.

Over a five-year period, the research team collected interview and focus group data in all phases of the research: Data collection began with community engagement work discussions that established principles for our research [7], followed by formative research to develop the intervention [39] and a process evaluation of the RCT implementation, and finally, exit interviews at the wrap up stage of the RCT [7,38]. Formal interviews and focus groups were transcribed verbatim. We kept field notes on informal discussions during site visits and the research process in general. We also conducted a participant satisfaction survey. Table 1 provides an overview of the complete data set whereas details of associated methodology were provided in previous publications.

A total of 34 interviews and 12 focus group discussions with a total of 142 participants were held in 6 communities over the period of 5 years (Table 2).

**Table 1:** Qualitative data analyzed in this study.

Method	Community engagement phase (pre-RCT^a^)	Implementation phase (RCT ongoing)	Close out phase (post-RCT)
Data collection tool	I-RREACH^b^ Engagement Tool [7] for Health Leaders SMS^c^ focus group discussion [39] with community members (potential RCT participants)	Process Evaluation Key Informant Interview Tool for Health Leaders Process Evaluation Key Informant Interview Tool for Health Care Providers	Process Evaluation Key Informant Interview Tool for Health Leaders Process Evaluation Key Informant Interview Tool for Health Care Providers SMS Close Out Evaluation Tool for Community RCT Participants Patient and provider impact of cell phone use–discussion group with health care providers and community RCT participants
Summary of data collection methods	Interviews Focus groups Site visit notes Patient/provider evaluation surveys	Provider interviews Site visit notes	Provider interviews Site visit notes Patient evaluation (satisfaction survey) Patient/provider interviews and discussion groups

^a^RCT: randomized controlled trial.

^b^I-RREACH: Intervention and Research Readiness Engagement and Assessment of Community Health Care.

^c^SMS: short message service.

**Table 2:** Interviews, focus groups, evaluations, and site visit notes.

Data collected		Total number		Total participants	
**Focus groups**		12		104	
	I-RREACH^a^		9		74
	SMS^b^		3		30
**Interviews**		34		38	
	I-RREACH		7		7
	Process evaluation		10		11
	Close out process evaluation		5		5
	Impact of cell phone discussion groups		12		15
**Survey evaluations**		233		—^c^	
	I-RREACH		49		—
	SMS close out questionnaires		184		—
Site visit notes		13		—	
Total focus group and interview participants		—		142	

^a^I-RREACH: Intervention and Research Readiness Engagement and Assessment of Community Health Care.

^b^SMS: short message service.

^c^Not applicable.

### Concept of Cultural Safety That Guided the Analysis

Cultural safety is a framework for understanding power differentials between health professionals and the Indigenous peoples they serve, and the negative impact of historical, social, and political power imbalances on health disparities [40]. It provides space for health care practitioners to explore the impact of these power imbalances on health while also respecting Indigenous goals of decolonization. In Indigenous health research, the privileging of Western epistemologies and methods in research over Indigenous knowledge and experiences is not only ineffective but also unethical [8,41]. Culturally safe research requires researchers to reﬂect on their tacitly held values, beliefs, and practices. Researchers and the organizations that support them must learn to identify, understand, and change routine practices, habits, or behaviors that create unsafe experiences for communities and participants [42]. Most importantly, it is the Indigenous research participants (not the researchers) who determine if a research project has been culturally safe and this must be reflected in the methodology [43]. In our analysis we therefore sought out narratives that illustrated cultural safety (or lack thereof) from the perspective of Indigenous research participants.

### Data Analysis

We utilized an inductive approach to the thematic analysis of the transcripts, with two researchers coding and categorizing the data for themes related to cultural safety, as expressed by participants. We then contextualized the emerging cultural safety themes around implementation issues, including the impact of the technology and delegation of tasks such as measuring patients’ blood pressure to nonregulated providers (also known as task shifting) [44]. We focused on understanding what factors led to a perceived fit (or lack of fit) with cultural safety based on the perspectives of Indigenous patients and staff. Our results were also informed by field notes which documented our learnings on cultural safety throughout the trial and were based on informal conversations at the community level. Once a preliminary analysis was completed, community-based Indigenous co-researchers provided feedback on the thematic analysis during several meetings and verified the final analysis through rigorous member checking.

### Ethics

Ethics review was completed by community-based First Nations REBs and university-based REBs. Community-based ethics review in First Nations communities included The Cree Board of Health and Social Services of James Bay, and the Manitoulin Anishinaabek Research Review Committee [34]. The study was formally approved by First Nations leadership through Band Council Resolutions. Academic ethics approvals include: (1) Queen’s University Health Sciences and Affiliated Teaching Hospitals Research Ethics Board (DMED-1603-13); and (2) Sunnybrook Health Sciences Centre Research Ethics Board (#182-2013).

### Trial Status

The RCT was completed December 2017 and outcomes are published [37].

## Results

### Summary

Based on our analysis, successful eHealth research in collaboration with Indigenous communities requires a focus on cultural safety that includes: (1) building a respectful relationship; (2) maintaining a respectful relationship; (3) good communication and support for the local team during the RCT; (4) commitment to co-designing the innovation; (5) supporting task shifting with the local team; and (6) reflecting on our mistakes and lessons learned or areas for improvement that support learning and cultural safety.

### Building a Respectful Relationship

#### Overview

Community staff linked cultural safety with the researchers’ commitments to building a respectful relationship based on open and transparent in-person meetings in the community. The focus on the community’s perspective, including each community’s unique cultural protocols, must be established during the community engagement and implementation process.

#### Face to Face Always [Has] the Best Results (Community B)

Staff emphasized the importance of meeting in person with the research team, including the principal investigator (PI).

The commitment to come here, I think that’s big.

Community E

Despite being a clinician in a busy hospital, taking time to visit each community on multiple occasions allowed the PI (ST) to learn directly from health staff, address concerns, and to provide background information about the research. The research coordinator (NP) was also a key participant in visits. In-person conversations established the rapport with the local team to facilitate trouble shooting of technology, recruitment, and clinical issues throughout the study. Other members of the team attended at the community level when possible.

#### Focus Was on Our Problems (Community B)

Cultural safety from the perspective of the participants involved focusing on community perceived issues as opposed to “researcher-knows-best” issues. For this, researchers required a basic knowledge of Indigenous determinants of health and the legacy of colonial policies [25]. Participants felt validated as researchers acknowledged colonial effects on Indigenous health by including a “discussion of multigenerational impacts” (Community B) on hypertension. Specific determinants of health that affected communities to different degrees, such as poverty, meant, “not everyone had cell phones” (Community B). The research team responded to this by providing simple phones to those in need so that everyone had the ability to participate in the project, which was important to community-based advisors.

#### Openness of the Researchers (Community D)

Taking the time for open dialogue and taking community perspectives seriously was one of the most often cited examples of cultural safety in DREAM-GLOBAL. Community staff appreciated the “comfortable environment to discuss the project openly and honestly” (Community D). They also stated that it was important to them that during meetings the “the atmosphere was good and friendly” (Community A), “and [community staff] felt at ease” (Community A) with the research team, who were “easy to talk to” (Community A). This encouraged local “people to share freely” (Community B) and “gain an understanding of the program and realizing there are many supports in place” (Community A).

The importance of addressing power imbalances was raised as participants stated their appreciation that the researchers were “non-judgmental…gentle and well-versed” (Community C) in working appropriately with Indigenous people. The community staff questions were taken seriously and “the research group went into detail” (Community A) about all aspects of the project, including concerns about confidentiality of health records, which were thoroughly addressed by the PI. This elicited the remark: “[you] made [it] very clear about confidentiality–Miigwetch (translation: thank you)” (Community B).

#### Cultural and Traditional Approach Was Taken Into Consideration (Community B)

Cultural safety is also about respecting and accepting the culture of the local clinics:

The team seems used to working with Indigenous communities. They accept our pace. They aren’t trying to turn this into a clinic in the South.

Community F

Staff liked that the “people leading were positive” (Community C), “their tone was relaxing and comforting” (Community C) and they “did not feel rushed” (Community C) in their work with the community. Cultural safety also came into play at the patient level, when the team realized that the SMS messages needed to be adapted to include traditional activities and foods based on focus group feedback [39]. From the researcher perspective, this was accomplished by asking community members to co-develop the text messages:

We got some great ideas for incorporating traditional foods and activities in an appropriate manner [into the intervention during our community visit]. For example, something like “Keep wild meat/foods healthy by boiling, broiling, stewing and limiting added fats…”

Field notes on community discussion during formative research in Community A

### Maintaining a Respectful Relationship

#### Their Visits to the Community Keeps Us Motivated (Community E)

Maintaining the relationship between the community and the researchers was an ongoing process and could not be reduced to a kick-off event. Participants explained that cultural safety in research required a focus on strengthening the collaborative relationship:

In First Nation communities, that’s huge, relationship building. The level of comfort is there [with the research team]. People come into the room and sit and talk when the DG team is here, so that means they feel comfortable. And then, when they have that level of comfort, they’re open to what you have to say.

Community E

Nurturing the relationship required that researchers were available to meet with all kinds of community members, leaders, and groups, not merely the gatekeepers.

I think it was really respectful. You met with the Elders, the band office, the CHRs (Community Health Representatives), so that was – yes, I think it just respects the basic collaboration criteria you have to do in…research with First Nations.

Community F

#### There’s a Feast for DREAM-GLOBAL (Community F)

The Indigenous cultural value of sharing is at the root of events such as community feasts. With support from the local staff, the DREAM-GLOBAL team hosted community feasts to share with the community as a whole, while also creating awareness about the project.

I think the feast is a good way also. I heard the community talking about “Oh yeah there’s a feast for DREAM-GLOBAL.” So there was some person there […] and she never heard before of the DREAM-GLOBAL study and she said ‘Oh that’s great, I also have high blood pressure.’ I’m happy to hear what you did, like the small teaching in front. […] I think it’s a [good] way to communicate.

Community F

Other communities also found feasts to be a culturally appropriate way to promote DREAM-GLOBAL.

Most people now know about it, especially since the Elders’ meal yesterday, there was quite a buzz yesterday, and now a lot of people are coming to check what’s going on.

Community E

#### You Tasted Our Traditional Food (Community F)

Participants emphasized the importance of positive interactions and that the openness of the researchers for engaging with the local culture and traditions also contributed to culturally safe research.

So the respect and wanting to learn about the culture was there I believe. […] In my opinion, certainly was there because I know you tasted our traditional food.

Community F

### Good Communication and Support for the Local Team During the Randomized Controlled Trial

#### The Relationship Was Very Open (Community F)

Community staff consistently provided feedback that the key to culturally safe research was the supportive communication with the DREAM-GLOBAL team, which helped to strengthen the relationship.

I think the relationship was very open. […] Any issues, you always address them. You’re always open to respond…

Community F

Any questions that arise, …[the researchers] are always available to answer our questions and provide us with assistance.

Community C

Timely access to the nurse research coordinator was considered an important aspect of DREAM-GLOBAL, especially the supportive troubleshooting.

I find the team is very supportive. If I need anything, I can quickly do a text message. I can get the information right away. I don’t have to wait.

Community D

We always feel that you are there to support us. There’s never been issues where we don’t receive the feedback that we need. It’s been excellent.

Community E

The tone of the interaction was also frequently mentioned:

Communications with the DREAM-GLOBAL team was all very pleasant and I feel very supported by them. […] They are approachable and it’s good to have good support. It’s important to have people who are understanding, friendly and encouraging.

Community C

Respect, openness, kindness, and support were clearly key qualities that the community staff valued in the communication.

### Commitment to Co-Designing the Innovation

#### Balancing Culture and Trial Design

The main challenge in co-designing a culturally safe intervention was finding ways to balance the clinical trial requirements with community culture. For example, early on we were told that a strict randomized trial where some participants received treatment and others placebo was not in line with Indigenous cultural values of respect and sharing. We therefore used “active versus passive” text messages, which resulted in a reduced-treatment arm as opposed to a no-treatment arm. However, it did not stop there, as the messages were not culturally safe.

#### They Want Us to Rephrase all SMS Messages (Field Notes)

Our evidence-based hypertension treatment text messages underwent rigorous testing for cultural safety in each of the participating Indigenous communities. Phrasing was important for cultural safety, as Indigenous patients interpret text messages within the context of their personal experience of oppression and racism in medical institutions. Indigenous community members taught the research team to avoid message content that could be perceived as paternalistic, fear-inducing, oppressive or authoritarian.

[Participants] want us to rephrase all messages that “compel”. So for example: “keep taking your meds as instructed….” This phrasing elicited a really emotional response and active resistance in our participants. “Don’t tell me what to do like I am a kid – offer us choices and reminders instead”. So these will need to be changed to “It is a good idea to take medications as indicated by your health care provider” or ‘Have you taken your meds today?’ etc.

Field notes in Community A

Many involved in the testing had strong dislikes for messages that would be acceptable to most Canadians. Cocreating a culturally safe version required unplanned formative research [39] but ensured that the messages were perceived as welcome and trustworthy. At close out, community participants reflected on the cultural appropriateness of the cocreated messages:

And they still, to this day […they] tell us, you know, “I’ve learned a lot from those messages that I wasn’t aware of before.”

Community F

I felt like somebody cared about my health. The messages were good reminders and were motivating.

Community F–SMS participant feedback questionnaire

Additional details, such as how many text messages patients were to receive and at what time of the day, were determined through dialogue with community staff. They identified an optimal frequency and timing, which was later validated as a good fit by participating patients:

Every couple of days when they get their text, it says something about the way they should be eating, lowering their salt intake. And they’re able to work on it for that week. It’s kind of like a reminder for them to keep on, you know, taking little baby steps. It’s a constant little gentle reminder, nothing too harsh but at the same time it turns into a huge positive outcome for them later on.

Community D

### Supporting Task Shifting With the Local Team

#### Feeling Very Closely Involved (Community F)

Staff felt it was important that the research team listened equally, and in a nonhierarchical manner, to incorporate input from all community team members:

There is a real partnership between the research team […] and the CHRs. They are very happy that [the PI] actually came to the community at the implementation of the study. The team regularly asks for the CHRs input etc. […] The CHRs love that they are feeling very closely involved in the project.

Community F

This emphasis on collaboration with all team members, including the nonregulated health care workers, was crucial when community health representatives took on new roles. Blood pressure monitoring or management was a task that shifted from nursing staff to community health representatives, which required culturally safe training and support to empower community health representatives to perform this new task:

At the beginning… we didn’t feel comfortable because I’ve never done it as a CHR, but as we progressed… in the study, I really looked forward to meeting once a month, the client, the participants…CHRs never had done the blood pressure check before so I felt like one of the health professionals. I felt proud of myself to do it.

Community F

…a personal success is becoming more comfortable with teaching and the physiology of BP and how it works. And I’ve become more confident the more I do it.

Community C

The RCT proved to be positive for personal and professional development of staff and it added to their recognition as they learned new capacities and current limits.

And at a staff level, we see people gaining knowledge and building capacity in how to manage hypertension and chronic diseases.

Community E-HD

The successful support for task shifting in turn supported recruitment for the study,

…one of the successes was it was easy to get people to buy into the program. We didn’t have to do too much in the way of PR, it was more word of mouth. People were really into leading a healthier lifestyle, it wasn’t hard for them to be swayed or we didn’t have to do a lot of teachings, I guess...[DREAM-GLOBAL] had a unique, innovative way of thinking about research studies, so that was one of the pluses about it. And our community was ready for it.

Community D

Within our primary care staff, it is good and we update each other every week on what we’ve done. And I always give an update on DREAM-GLOBAL to the staff, and that’s why I get a lot of support and referrals.

Community C

Some communities had slow recruitment periods, but the staff had become invested and persevered:

Initially it was challenging, then it seems all of a sudden there was a lot of interest. […] It’s all about timing. The right time, the right place. […] We saw at the beginning, we were able to get quite a few, then it was quiet, then we had an opportunity again to get more people enrolled.

Community E

Many felt that the expansion of the community health representative role was sustainable.

So for me the biggest success is to show what the CHR can do within their power and I think that’s going to leave traces for the next years that we’ll say well we know that you can do it and I’m sure they want to do it as well.

Community F

### Reflecting on Our Mistakes

#### Need for Reflexivity

Cultural safety in research required reflexivity about the effects of the project on participating communities, staff, and patients. Reflecting on DREAM-GLOBAL, our focus on cultural safety meant that many things went well but also that we learned from our mistakes.

#### In for a Week, Out for a Week, You Know? (Community F)

We attempted to adhere to community collaboration, but the health system is different in each First Nation community and understanding the mix of provincial and federal services and permanent, contract, and visiting staff can be challenging for visiting researchers. In one community, we had failed to notice that the functioning of the local team was fractured by staff turnover, and lack of integration of contract and community staff. Consequently, we missed the opportunity to consult with key members in the community at the beginning, which stymied the integration of the eHealth intervention within the local health services. Later we realized that we had to orient many more health care providers, including those who were in the community only intermittently.

I would have wanted the doctors to be more involved but…they couldn’t really be part of it because they’re always in and out of the community because they have other communities to [be] responsible [for].…CHRs have to keep reminding the MDs to refer and still they don’t do so enough.

Community F

One of the major problems was that management was not included in the community discussion as to whether to accept to participate in the DG study or not. [The director] feels like the doctors went over her head.

Community F

Our failure to understand who could speak on behalf of the community and who could decide to integrate the eHealth innovation into the services also lead to an overestimation of hypertension as a health priority.

I think people just forgot…blood pressure is something not in the first priorities. Here, I think it’s more like diabetes.

Community F

#### I Felt a Little Bit Out of the Loop (Community F)

The effect of our oversight snowballed and affected important aspects of the implementation of DREAM-GLOBAL, including recruitment and communication:

…doctors… are coming in and out of this health care system all the time. So were we catching all the [patients] who would be open to take part in the project? Probably not… Maybe not everybody knew to ask, you know?

Community F

she was told by one of the MDs that the nurses would have nothing else to do but to refer the patients to the CHRs, but… the CHRs would ask her if she had discussed the study with the patient prior to the referral; there seemed to be a misunderstanding in each person’s role.

Community F

The shifting of new tasks to the community health representative was also initially much less accepted at this site as we were struggling with a culturally safe approach.

I get the feeling that when we refer to the CHR, some of [the patients] may feel like we’ve just passed them off to somebody else. […] So a lot of times they don’t want to go see the CHR and sometimes maybe it’s because they have like personal issues with some of the CHRs, I don’t know. It’s a small community, you know, family issues between families.

Community F

However, on the positive side, using our own methods of reflection and face to face visits to resolve our rocky start, we achieved a satisfactory level of implementation in the end, especially after spending more time with the community health representatives.

At the beginning it was slow but once we got the awareness and when we did that blood screening at the commercial center […] then the people were just coming, like flocking to our screening.

Community F

## Discussion

### Primary Findings

At the onset of the RCT, our team was committed to cultural safety in our research process and seeking the perspective of the community in all phases of the project. We often succeeded with our approach, but despite good intentions we sometimes fell short. Carving out time for reflection on these miscalculations with community members and as a research team was important to transform these experiences into learning opportunities and to resolve misunderstandings.

**Figure 1:**
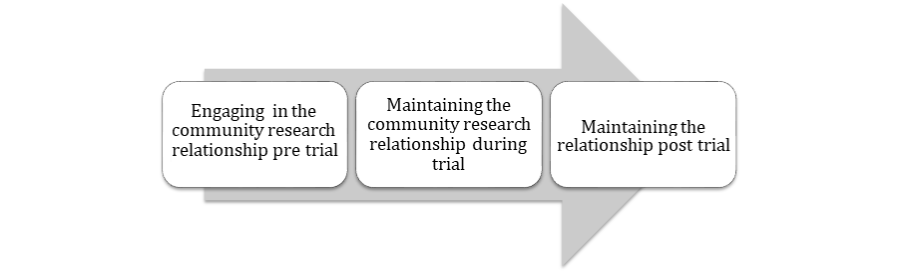
Research relationship stages.

Listening to Indigenous community staff, elders, and patients during our community visits, we discovered that in order to prepare for clinical trial research the most significant undertaking is tending to the relationship between the researchers and the community at various stages of the work (Figure 1).

**Figure 2:**
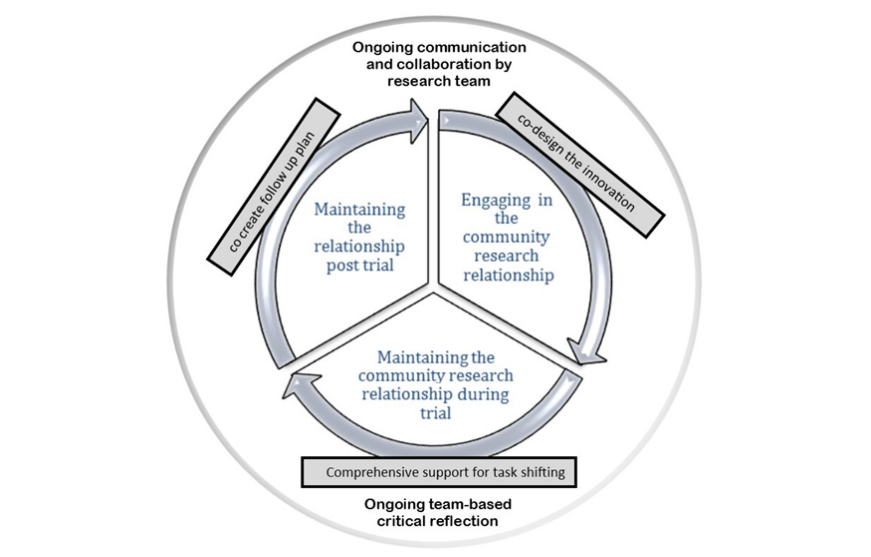
Maintaining research relationships.

The relationship building starts with an engagement process, where researchers and communities learn about topics such as community issues and cultural and research protocols and come to a consensus about expectations for the trial phase. Maintaining research relationships after the trial may include cocreating a plan for community presentations, report writing, advocacy for sustainability, and planning for future collaborations. In this case the relationship becomes circular, as demonstrated in Figure 2. These findings are conceptually in line with established ethical practices in Indigenous research [8,32,45,46], as well as supported by our previous research [7,27,39,44].

The findings in this study are tailored to pragmatic clinical trial research and to eHealth research with Indigenous communities, and they illustrated, with selected narratives, that researchers can gain a better understanding of various Indigenous perspectives related to RCT practices and methodologies. The narratives underscore the important cultural value of relationship building shared by all participating Indigenous groups, and the role that researchers’ commitment, community immersion, good communication, and supportive attitude plays in strengthening their relationship with the community. Electronic Health requires a thorough assessment of how the intervention will affect local workflow in a way that is empowering to the community, with task shifting one important way to achieve this goal. Finally, time for critical reflection with the community representatives and within the research team to understand good practices and mistakes are important aspects of culturally safe research.

Building on the literature and the results of our research with six culturally diverse Indigenous communities, we formulated wise practices for cultural safety in RCT and eHealth research. We do not claim that these are exhaustive practices, nor that they apply exactly as stated to all Indigenous communities. Instead, we invite researchers and Indigenous community partners who collaborate on RCT and eHealth research to review these practices as a starting point. We believe most points will resonate, however, some may need to be collaboratively added or modified to the unique characteristics of each Indigenous community and the corresponding research project.

### Wise Practices for Culturally Safe Randomized Clinical Trial Research

#### Focus on Researcher Readiness

Researchers need to learn about Indigenous issues and take this up as part of life-long learning. This process may include completing training (including online) on cultural safety, self-reflection, ethical space dialogue, Indigenous culture, history, and treaties to gain capacity in this area [7,35]. Course work should be complemented with experiential work and immersion in Indigenous culture and communities whenever possible.

Changing the research lexicon, from investigator to researcher and from trial to study may also be helpful. These words are less charged with meanings that can lead to reduced trust. Learning about the specific communities involved is also important.

#### Reflective Research Practice

Reflection is a key component of culturally safe care, and researchers should participate in regular ongoing reflection on cultural safety in research [40]. Similarly, critical reflection should happen on a personal level, as a research team, and as a project team together with the local Indigenous team. Open dialogue should support this activity and translate into adjustments towards more culturally safe approaches as needed. It may be helpful to reflect in pairs, so that if the meaning is not fully understood dialogue can lead to increased understanding.

#### Principal Investigators and Study Coordinators Need to Build a Relationship With Communities

Both the principal investigators and study coordinators should spend significant time in face-to-face meetings in the community during all phases of the project to build a respectful rapport and answer all questions openly and honestly in an informal manner. This will ensure that both the key decision makers and support personnel are aware of and understand community wishes, concerns, and opportunities related to the project. Meetings should include local clinical champions who should be well integrated in the community [7].

#### Trial Should Include Some Benefits for all Participants

A treatment versus no treatment control design is often deemed to be culturally inappropriate or unethical from the perspective of Indigenous world views. Trial design should allow for benefits to the control group by providing, for example, reduced treatment in a pragmatic trial, or delayed treatment (step wedge design trial). Small gifts to acknowledge appreciation for participation are welcome (and often a cultural practice), particularly practical ones such as healthy food boxes or gift cards for local produce retailers [27].

#### All Phases of the Electronic Health Intervention Are Co-Designed

The health issue, messages, graphics, research process, and all aspects of the technology must be acceptable to participants. This requires formative research to co-design the intervention in collaboration with the Indigenous team members, to ensure that potential adaptations during implementation and follow up after the trial are completed so that there are lasting benefits to the community [39,44]. While there is an understanding that there are health disparities, true co-design will likely focus on incorporating a strengths-based approach to the health issue and will build on Indigenous culture, community, and resilience.

#### Technology and Research Support

Technology support will be required to troubleshoot, whereas research support is needed for questions related to recruitment, blinding, etc. One key contact person who has established a relationship with the community is needed to respond quickly to community staff’s questions. This person may not have all the answers but should be well connected with all members of the research team so they can get the answers in a timely fashion [38].

#### Support for Task Shifting

The task shifting should support local goals for self-sufficiency in community health and community empowerment. Changes in roles require that researchers learn to understand the local work dynamics and advocate for acceptable shifting of tasks within the local health system. To achieve this, training and supporting the community staff who carry out the new task, as well as the managers, is necessary.

#### Research Budgets Must Reflect the Nature of Community-Based Participatory Research

Budgets need to cover researcher travel to communities, community-based collaborator travel to urban meetings or conferences, and culturally appropriate local hospitality, honoraria, or gifts. It must also cover post study knowledge transfer to ensure that community members feel that they have benefitted as much as possible from the new learnings from the project. Timelines for spending funds requires flexibility to be respectful of local priorities and competing commitments to avoid overburdening the community workers.

### Limitations

Our work was limited to six First Nations communities, thus there are limitations related to the generalizability of the work. However, as many of the themes were found in these culturally diverse communities that included Cree, Mi'kmaq, Pottawatomi, Ojibwa and Odawa tribes, it is likely that the criteria for cultural safety in research will strongly resonate with many other Indigenous peoples in Canada and in other countries.

### Conclusions

Based on evaluation data collected over the five years of the DREAM-GLOBAL RCT, we found that there were important cultural safety considerations in Indigenous eHealth research. Building on the perspectives of Indigenous staff and patients, we gleaned wise practices for RCTs and eHealth research in Indigenous communities.

Cultural safety in eHealth research is dependent on: the quality of community engagement and collaboration on all phases of the research; sustained relationship building; respectful communication; timely implementation support; a commitment to co-design the innovation in collaboration with Indigenous partners, and support for culturally appropriate task-shifting. Finally, reflecting and learning from mistakes is needed to ensure cultural safety.

## Abbreviations

BP: blood pressure
CHR: community health representative
DREAM-GLOBAL: Diagnosing Hypertension-Engaging Action and Management in Getting Lower Blood Pressure in Indigenous Peoples and Low- and Middle- Income Countries
eHealth: electronic health
I-RREACH: Intervention and Research Readiness Engagement and Assessment of Community Health Care
mHealth: mobile health
PI: principal investigator
RCT: randomized controlled trial
SMS: short message service
